# Editorial: Allogeneic haematopoietic stem cell transplantation for children with acute lymphoblastic leukaemia in the era of immunotherapy

**DOI:** 10.3389/fped.2022.959471

**Published:** 2022-08-16

**Authors:** Christina Peters, Adriana Balduzzi, Peter Bader

**Affiliations:** ^1^Stem Cell Transplantation Unit, Department of Pediatrics, St. Anna Children's Hospital, Children's Cancer Research Institute, Medical University of Vienna, Vienna, Austria; ^2^Clinica Pediatrica Università degli Studi di Milano-Bicocca, Fondazione MBBM, Monza, Italy; ^3^Division for Stem Cell Transplantation, Immunology and Intensive Care Medicine, Department for Children and Adolescents, University Hospital, Goethe University, Frankfurt, Germany

**Keywords:** paediatric, haematopoietic stem cell transplantation, chemotherapy, ALL-acute lymphoblastic leukaemia, forum, total body irradiation (TBI)

## Introduction

**Figure 1:**
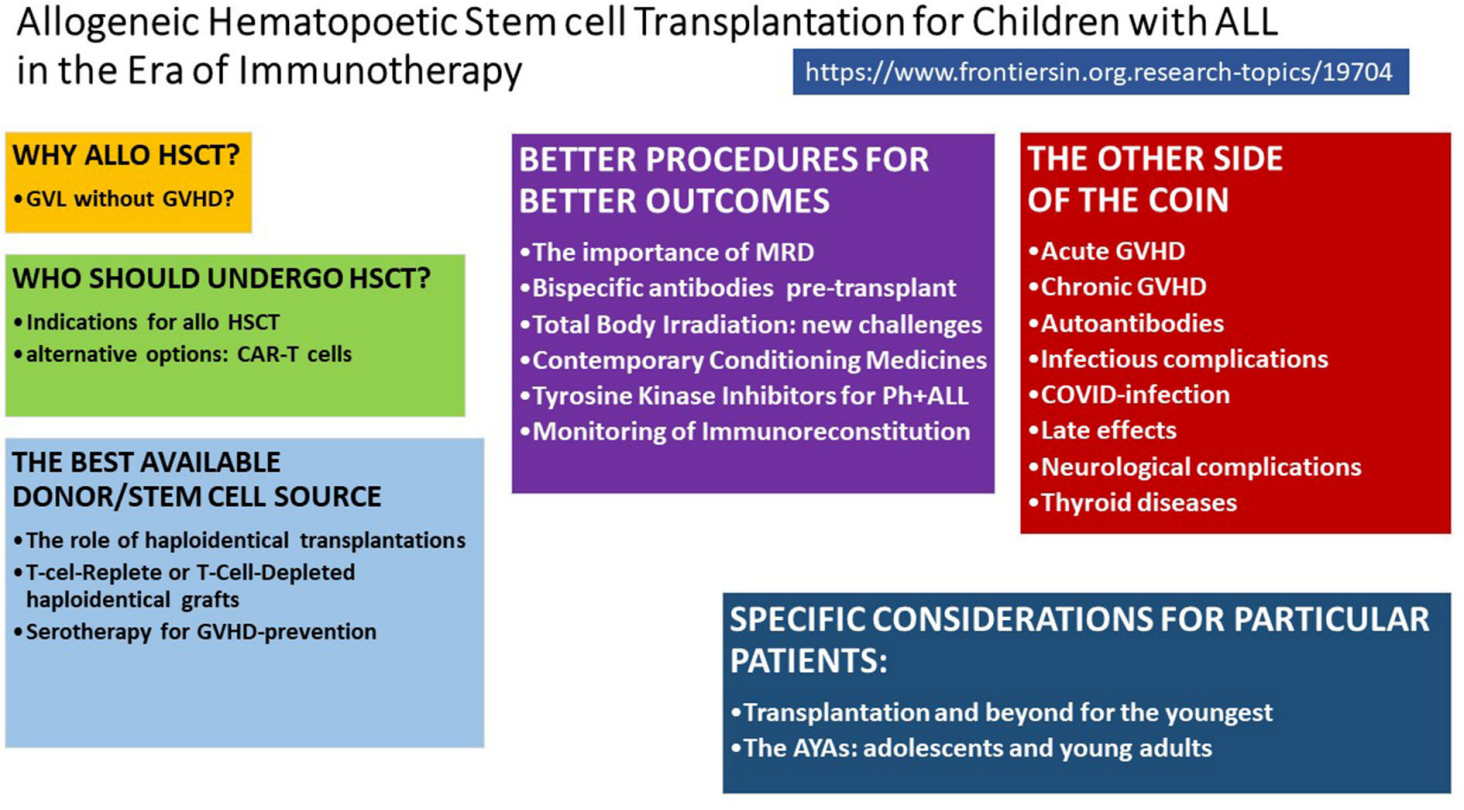
Tools and challenges to cure high-risk ALL.

Acute lymphoblastic leukaemia (ALL) is the most common paediatric cancer; ~60% of ALL cases occur in children and adolescents younger than 20 years (1). In the 1960s, it was first reported that childhood ALL was no longer incurable. Since then, outcomes for children and adolescents with ALL have improved remarkably thanks to new diagnostic technologies, effective administration of conventional chemotherapy, and provision of better supportive care (2). Allogeneic haematopoietic stem cell transplantation (HSCT) became the most commonly applied immunotherapy and the standard of care for children with ALL that was at high relapse risk or had relapsed. HSCT has supported more than 70% of patients in this high-risk group becoming long-term survivors (3). The most frequent cause of treatment failure is relapse; the risk of post-transplant relapse is influenced by conditioning regimen, remission status at transplantation, and donor type (4) (Figure 1).

Over the last decade, a new era of immunotherapy began. Innovative strategies incorporating immunotherapy became available as salvage therapies for the highest risk patients and possible additions to standard of care to further improve long-term leukaemia-free survival with fewer side effects (5). Further improvements have included a reduction of minimal residual disease (MRD) pre-transplant (6), the substitution of toxic chemotherapy with bispecific antibodies, replacement of HSCT with chimeric antigen receptor (CAR) T-cell therapy (7), improved transplant strategies for specific groups, including infants (8) and adolescents and young adults (AYA) (9), and innovative prophylaxis and treatments for acute and chronic graft-vs.-host disease (GvHD). Furthermore, therapeutic drug monitoring and dose adjustment (10) and novel radiation techniques might enable individualised therapies.

In this manuscript collection, more than 100 outstanding experts discuss the state of the art and the most promising tools for preventing relapse of paediatric ALL and reducing transplant-associated side effects without jeopardising efficacy.

## Why allogeneic HSCT?

The review by Rozmus et al. addresses the question of whether it is possible to separate the graft-vs.-leukaemia (GvL) effect from GvHD. To control re-occurrence of malignant ALL-blasts, alloreactive donor-T-cells against recipient leukaemia need to mature and expand after HSCT. The mechanisms of GVL and acute and chronic GvHD are similar and thus limiting GvHD-associated inflammation is warranted. Graft manipulation, but also more specific cell therapies and pharmacological strategies in combination with myeloablative conditioning enable nowadays-powerful transplantation modalities to eradicate ALL with low risk for dangerous T-cell-reactions against healthy recipient tissues.

## Who should undergo HSCT in 2022 and beyond?

To define current indications for allogeneic HSCT and possible alternative options, Truong et al. summarise the strategies of the major front-line study groups. For patients in first remission, genetic aberrations and response to induction and consolidation chemotherapy are the most powerful tools to identify early those patients at high relapse risk in need of an HSCT. In the relapsed setting, time and site of relapse and response to rescue chemotherapy drive the decision-making process. Patients with T-cell ALL almost invariably need consolidation with allogeneic HSCT. Specific algorithms are proposed for Philadelphia-chromosome (Ph)-positive patients, as detailed by Vettenranta et al. Patients who do not achieve morphological remission need novel (immune-) therapeutics for preparation to transplant.

Buechner et al. address the question of for whom CAR T-cell therapy might be an option to substitute allogeneic HSCT and in which situations it might be a bridge to HSCT. They describe the outcomes of CAR T-cell therapy in B-cell precursor (BCP) ALL and discuss factors associated with favourable outcome and limitations to this therapy. They identify knowledge gaps in the use of CAR T-cell therapy, especially the unknown late effects and long-term efficacy, as well as the lack of robust phase III studies comparing standard of care (including HSCT) to CAR T-cell therapy.

## The best available donor and stem cell source

Historically, the best stem cell donor for allogeneic HSCT was considered a human leukocyte antigen (HLA)-identical sibling and the best stem cell source was considered bone marrow. However, for the last two decades, the outcome of HSCT from an HLA-compatible unrelated donor, with compatibility defined by high-resolution typing and after intensified GvHD prophylaxis, has been comparable to HSCT from an HLA-identical sibling. Furthermore, survival after HSCT using a graft from bone marrow vs. peripheral blood stem cells is comparable despite a controversial increase in GvHD.

Approximately 20% of the patients in need for an allograft are candidates for transplantation from donors who are partially HLA mismatched. Rahman et al. review the progressive experience with use of haploidentical family donors for the transplantation of children with ALL. Beside the benefits of HLA diversity, the available literature on donor selection within the family are summarised and recommendations are provided. Furthermore, the ethical considerations of using minors as stem cell donors are discussed.

Kleinschmidt et al. provide an in-depth review of the pros and cons of manipulation techniques for haploidentical grafts. Two main approaches to prevent graft rejection and life threatening GvHD are currently applied: *ex vivo* T-cell depletion (TCD) and *in vivo* T-cell suppression. Published data are mainly limited to single centres or single countries, especially for children with ALL.

The drawback of highly effective GvHD prophylaxis, with either *in-vitro* or *in-vivo* approaches, is a slower T-cell immune reconstitution, which leads to a higher infection risk. Keogh et al. review the available literature on the different serotherapeutic agents used for GvHD prophylaxis and provide perspectives on the optimization of dosing using therapeutic drug monitoring and population-based pharmacokinetic modelling.

## Better transplant procedures for better outcomes

MRD prior to and after HSCT is a major factor for outcome, with high MRD burden negatively correlated with post-transplant survival. Merli et al. review and discuss quantification methods for MRD (polymerase chain reaction, fluorescence-activated cell sorting, and next-generation sequencing), the threshold of MRD, as well as possible pre- and post-transplant intervention strategies, including pre-transplant therapy intensification and post-transplant immunological interventions.

One of the most effective off-the-shelf immunotherapies is blinatumomab, a bispecific T-cell engager (BITE). Kallay et al. review blinatumomab alongside antibody–drug conjugates, such as inotuzumab ozogamicin, and monoclonal antibodies, such as daratumumab. The role of these novel agents in reducing pre-transplant MRD and their potential to reduce toxicity, compared with traditional chemotherapy, are described. Furthermore, their efficacy for post-transplant relapse and possible beneficial effect for vulnerable patient groups, such as infants and patients with trisomy 21, are discussed.

As recently shown in a large, prospective randomised trial (FORUM – For Omitting Radiation Under Majority age), total body irradiation (TBI) in combination with etoposide is superior to two different chemoconditioning regimens consisting of fludarabine and thiotepa in combination with either busulfan or treosulfan (4). The trial confirmed the TBI-based regimen as the gold standard myeloablative conditioning regimen for children ≥4 years and young adults with ALL. However, despite the reduction of transplant-associated acute complications, HSCT and TBI are still associated with long term complications, including hormonal impairment, infertility, cataracts and risk for secondary malignancies.

Hoeben et al. present the state of the art of TBI use, considering dose, fractionation, dose rate and set-up performance. Limitations and novel strategies for improvement are extensively discussed including the need for assessing the impact of innovative modalities and ultimately the harmonisation of irradiation techniques.

Despite the striking outcome of the FORUM trial, several late effects have to be kept in mind. Less toxic, irradiation-free, myeloablative conditioning regimens that are suitable alternatives to TBI-based regimens should be sought. Hassine et al. provide a comprehensive update on the use of targeted drug monitoring (TDM) to adjust the dosing and control of conditioning medicines, particularly busulfan, treosulfan, fludarabine and clofarabine to the individual patient. They also give insights into busulfan pharmacogenomics and discuss alternatives to the classical 4-day administration schedule. Especially for patients below the age of 4 years, treosulfan TDM and individualised dosing of immunosuppressants such as ATG might optimise chemotherapy-based conditioning therapies.

## How to prevent, diagnose, and treat transplant-associated complications

### Acute GvHD

Although the incidence of severe acute GvHD following HSCT is lower in children compared to adults, acute GvHD is still a driver for early complications. Woelfl et al. review current prophylaxis and treatment options for acute GvHD in children with ALL. Not only the pathophysiology of aGvHD is different, but also the incidence of comorbidities is lower in children. Due to better organ functions, side effects of drugs are better tolerated and the thymic function might allow better donor-T-cell recovery. The growing importance of specific biomarkers and the use for optimised prophylaxis and treatment of acute GvHD are proposed.

### Chronic GvHD

Preventing and managing chronic GvHD is of the utmost importance for young patients because it is the most devastating complication of HSCT, not only leading to irreversible tissue damage but also dramatically affecting quality of life. Sobkowiak-Sobierajska et al. cover current treatment options for chronic GvHD, including topical and systemic treatments and immunomodulatory approaches, in the attempt to balance immune reconstitution, the risk of leukaemic relapse and risk of infection.

Lawitschka et al. present new data on the role of autoantibody expression in paediatric HSCT recipients. The data show that autoantibody profiles are not suitable biomarkers for diagnosing chronic GvHD in children or for predicting its severity, disease course and outcome. However, the study identified a significant immune dysbalance associated with *active* chronic GvHD in paediatric patients with ALL who underwent HSCT. This echoes results of adult studies and are in line with previous results (11–13).

### Immune reconstitution and risk of infection

Delayed immune reconstitution is associated with an increased risk of serious infection. Yanir et al. discuss the pattern of immune reconstitution after HSCT.

Bacterial, fungal and viral infections are still a major cause of serious complications after HSCT, mainly in the first year post transplant and especially in patients with long-lasting chronic GvHD. Zajac-Spychala et al. review progress in the diagnosis, prophylaxis and treatment of infectious complications and recommendations for children with ALL after allogeneic HSCT. They also focus on vaccination policies post-transplant and on the development of individualised approaches to antimicrobial prophylaxis and empirical therapy.

Zubarovskaya et al. contribute to the topic of viral infections a case report of a patient with chronic GvHD and severe ARDS due to coronavirus 2 (SARS-CoV-2) infection. Surprisingly, despite severe lymphopaenia, the patient developed specific antibodies and cleared not only the infection but also survived the pulmonary complication without severe organ damage.

### Late effects

Thanks to improved HLA-typing- and –matching techniques, better infection-prevention and prophylaxis and treatment of acute and chronic GVHD, many patients become long-term survivors after allogeneic HSCT. Thus, aftercare, especially for children who were transplanted at young age, becomes an essential part of stem cell transplantation protocols. Specialised teams have to support their patients and initiate timely diagnosis and treatment of organ dysfunction and consequences of irradiation, immunosuppression and drug toxicities.

Three manuscripts in this supplement focus on these important topics.

Diesch-Furlanetto et al. review late effects after allogeneic HSCT for paediatric AL. The authors give a comprehensive description of organ-specific late complications, psychosocial consequences and quality of life, and how to manage transition to adult services. Comprehensive reports of their individual disease and the given treatment alongside with checklists and recommendation for healthy lifestyle enable a safe transfer from paediatric to specialists for AYAs.

The majority of the literature on acute and long-term neurological complications is from studies performed in adults. Gabriel et al. reviewed data available for paediatric ALL. However, the risks of acute neurological symptoms, such as seizures or encephalopathy (including posterior reversible encephalopathy syndrome)—which can be associated with infections, methotrexate, busulfan and fludarabine use—and peripheral neuropathy—which can be associated with vincristine and calcineurin inhibitor use—are relatively high with estimates at 5 and 10%, respectively. In addition, the long term effects and late complications after cranial irradiation and especially myeloablative total body irradiation need special attention. For the quality of life of young patients, correction of hormonal dysbalance, neurocognitive impairment and the timely diagnosis of secondary brain tumours, are essential.

The third manuscript on the topic of late effects deals with thyroid complications after TBI for paediatric ALL. The toxic effect of TBI is known, yet data on the role of immunological dysregulation and chronic GvHD are scarce. Zubarovskaya et al. studied functional and structural thyroid disorders in 97 paediatric ALL patients after TBI-based conditioning for HSCT. They correlate their findings with basic characteristics of patients and donors and occurrence of chronic GVHD and found a high proportion of immunological dysregulation and thyroid complications with need for hormonal replacement and monitoring.

## Special considerations for particular patient groups

### Adolescents and young adults (AYAs)

It is well-known that young adult patients with ALL have better survival chances if they are treated according to paediatric protocols. Whether this is also true for the HSCT-setting was not proven in prospective trials.

Calvo et al. summarised the key findings of recent studies on treatment approach and outcomes in AYAs after HSCT. They describe the differences between paediatric and adult transplantation centres and the specific considerations for patients beyond 14 years of age. Especially the growing consequences of infertility and possible future solutions are addressed.

### Infants and young children

Infants diagnosed with ALL have a poorer overall and event free survival with contemporary chemotherapy. Especially the group with KMT2A gene rearrangement, high leucocyte count at diagnosis and disease onset in the first 6 months of life have dismal outcome and allogeneic HSCT might be the best available treatment option. (REF) Balduzzi et al. review the contemporary strategies for HSCT in children <4 years, from conditioning regimens and additional immunological treatment modalities, including bispecific antibodies. The goal is to reduce pre-transplant toxicity and to lower the leukaemic load to increase event free survival, particularly in the youngest age group.

## Summary

In conclusion, HSCT remains the most effective approach to treating ALL in children and adolescents at the highest risk of relapse. Whether innovative strategies will improve to the extent that they may substitute HSCT in most cases and so yield fewer and less-severe complications and sequelae remains to be assessed. Improvements in novel cell therapeutics are also a topic of future research.

## Author contributions

All authors listed have made a substantial, direct, and intellectual contribution to the work and approved it for publication.

## Conflict of interest

The authors declare that this editorial was written in the absence of any commercial or financial relationships that could be construed as a potential conflict of interest.

## Publisher's note

All claims expressed in this article are solely those of the authors and do not necessarily represent those of their affiliated organizations, or those of the publisher, the editors and the reviewers. Any product that may be evaluated in this article, or claim that may be made by its manufacturer, is not guaranteed or endorsed by the publisher.
